# Impact of the COVID‐19 pandemic on the in‐hospital diagnostic pathway of breast and colorectal cancer in the Netherlands: A population‐based study

**DOI:** 10.1002/cam4.6861

**Published:** 2024-01-10

**Authors:** Wouter Wolfkamp, Joyce Meijer, Jolanda C. van Hoeve, Felice van Erning, Lioe‐Fee de Geus‐Oei, Ignace de Hingh, Jeroen Veltman, Sabine Siesling, the COVID and Cancer‐NL Consortium

**Affiliations:** ^1^ Department of Research and Development Netherlands Comprehensive Cancer Organisation (IKNL) Utrecht the Netherlands; ^2^ Department of Health Technology and Services Research University of Twente Enschede the Netherlands; ^3^ Department of Surgical Oncology Catharina Cancer Institute Eindhoven the Netherlands; ^4^ Department of Radiology Leiden University Medical Center (LUMC) Leiden the Netherlands; ^5^ Biomedical Photonic Imaging Group University of Twente Enschede the Netherlands; ^6^ Department of Radiation Science & Technology Delft University of Technology Delft the Netherlands; ^7^ Department of Knowledge and Advice Netherlands Comprehensive Cancer Organisation (IKNL) Utrecht the Netherlands; ^8^ Department of Epidemiology, GROW‐School for Oncology Reproduction Maastricht University Maastricht The Netherlands; ^9^ Department of Radiology ZGT Almelo the Netherlands

**Keywords:** breast cancer, colorectal cancer, COVID‐19, diagnosis, diagnostic pathway, diagnostic procedures, lead time, population based

## Abstract

**Background:**

In the Netherlands, the COVID‐19 pandemic resulted in a temporary halt of population screening for cancer and limited hospital capacity for non‐COVID care. We aimed to investigate the impact of the pandemic on the in‐hospital diagnostic pathway of breast cancer (BC) and colorectal cancer (CRC).

**Methods:**

71,159 BC and 48,900 CRC patients were selected from the Netherlands Cancer Registry. Patients, diagnosed between January 2020 and July 2021, were divided into six periods and compared to the average of patients diagnosed in the same periods in 2017–2019. Diagnostic procedures performed were analysed using logistic regression. Lead time of the diagnostic pathway was analysed using Cox regression. Analyses were stratified for cancer type and corrected for age, sex (only CRC), stage and region.

**Results:**

For BC, less mammograms were performed during the first recovery period in 2020. More PET‐CTs were performed during the first peak, first recovery and third peak period. For CRC, less ultrasounds and more CT scans and MRIs were performed during the first peak. Lead time decreased the most during the first peak by 2 days (BC) and 8 days (CRC). Significantly fewer patients, mainly in lower stages, were diagnosed with BC (−47%) and CRC (−36%) during the first peak.

**Conclusion:**

Significant impact of the COVID‐19 pandemic was found on the diagnostic pathway, mainly during the first peak. In 2021, care returned to the same standards as before the pandemic. Long‐term effects on patient outcomes are not known yet and will be the subject of future research.

## 1 BACKGROUND

Late 2019, a new infection known as COVID‐19 disease was identified in Wuhan, China.^1^ The coronavirus spread quickly, became a worldwide problem and impacted cancer care. Delays in screening, diagnosis and treatment were observed which may result in an increase in cancer‐related deaths in the future.^2^, ^3^, ^4^ The infections in the Netherlands started in the south in February 2020. In March 2020, the first measures against the coronavirus were taken in the Netherlands with temporary halt of the population‐based screening programs for BC and CRC and societal measures.^5^, ^6^ People were advised to visit the general practitioner (GP) only in case of severe complaints and referrals to the hospital were postponed.^5^ Fewer diagnoses and treatments were performed and surgeries were postponed.^7^, ^8^ In case of a shortage of capacity, patients were transferred to other hospitals.^6^ After temporarily halting population screening from mid‐March 2020 to mid‐May 2020, screening was restarted gradually. Mid 2021, BC screening capacity was 85% and CRC screening had the same capacity in October 2021 as in the years before the pandemic.^9^

Until now, studies mainly focused on the first months of the pandemic or focused on treatment, follow‐up and the prediction of long‐term impact. Few studies focused on the impact of the COVID‐19 pandemic on cancer diagnosis. Some studies revealed the impact of the pandemic on CRC^10^ or the impact on cancer care in general.^4^ A study revealed that fewer and delayed referrals possibly influenced the diagnostic pathway in the hospital.^11^ Another study revealed a postponed start of cancer treatment and patients diagnosed with poorer patient and tumour characteristics during the pandemic.^12^

No studies are currently published that focus on the in‐hospital diagnostic pathway. As this may have impact on the treatment and subsequent outcome of these patients, the objective of this study was to determine the impact of the COVID‐19 pandemic on the in‐hospital diagnostic pathway until start of therapy by analysing the diagnostic procedures performed per patient and the time to start of therapy. This study provides a more complete understanding of cancer care during the pandemic and lessons can be learned to maximise the quality of cancer care during any subsequent pandemic.

## 2 METHODS

### 2.1 Data collection

This study is a retrospective cohort study, based on data from the Netherlands Cancer Registry (NCR) and Dutch Hospital Data (DHD). The data set consists of data on patient and tumour characteristics from the NCR (e.g. age, sex, type of cancer, stage of disease, region in the Netherlands) and data on the diagnostic procedures from DHD (e.g. diagnostic procedure performed, date of diagnosis, date of first diagnostic procedure, date of start therapy).

### 2.2 Patients

Patients aged 18 years or older diagnosed with primary BC or CRC during the period from January 2017 to July 2021 were included in this study. Data from DHD and the NCR were probabilistically linked on patient levels using the patient number, date of birth, sex and postal code. Diagnostic procedures more than 6 months prior to the date of diagnosis were removed to avoid including non‐cancer‐related diagnostic procedures in the analysis. Patients without known hospital diagnostic procedures, an unknown start the date of therapy or a start date of therapy before date of diagnosis were excluded.

### 2.3 Definitions

#### 2.3.1 Periods

The study period between January 2020 and July 2021 was divided into six periods based on the severity of the COVID‐19 pandemic for which we used hospitalizations due to COVID‐19 in the Netherlands as a proxy. Period A covers weeks 1–11 of 2020 (i.e. pre‐COVID), period B weeks 12–20 of 2020 (i.e. first peak), period C weeks 21–41 of 2020 (i.e. first recovery), period D weeks 42–53 of 2020 (i.e. second peak), period E weeks 1–20 of 2021 (i.e. third peak) and period F weeks 21–30 of 2021 (i.e. second recovery). For comparison, data for 2017–2019 were divided accordingly. The second and third peaks are divided into two periods to analyse 2020 and 2021 separately.

#### 2.3.2 Age categories

Patients were categorized into age categories, based on the age categories for population screening for BC and CRC: 50–75 and 55–75, respectively. Therefore, patients with BC were grouped into ages <50, 50–75 and >75 years and patients with CRC were grouped into ages <55, 55–75 and >75 years.

#### 2.3.3 Sex

For BC, only females were included. For CRC, males and females were included.

#### 2.3.4 Stage of disease

Stage of disease was divided into stages 0–4 for BC and 1–4 for CRC, based on the TNM classification (8th edition).^13^ Pathological stage was used if available. When pathological stage was unknown, the clinical stage was used. Any remaining unknown stage was classified as stage X.

#### 2.3.5 Region

Patients were categorized into a region, based on the hospital where a patient was diagnosed. In total, the Netherlands was divided into five regions,^14^ these are the north (Friesland, Groningen, Drenthe), the middle east (Overijssel, Flevoland), the middle (Utrecht, Gelderland), the west (Noord‐Holland, Zuid‐Holland, Zeeland) and the south (Noord‐Brabant, Limburg).

#### 2.3.6 Diagnostic procedures

Per type of cancer, different diagnostic procedures were analysed based on common diagnostic procedures per type of cancer.^15^ Diagnostic procedures were divided into mammography, ultrasound, PET‐CT and CT for BC and ultrasound, endoscopy, CT, MRI and PET‐CT for CRC.

#### 2.3.7 Lead time

The lead time of the diagnostic pathway per patient was calculated as the time between the first diagnostic procedure and the start of therapy. When the date of first diagnostic procedure was missing, the date of pathologically confirmed diagnosis was used as the date of first diagnostic procedure.

### 2.4 Statistical analysis

Periods during COVID‐19 were compared to the same periods in 2017 to 2019. BC and CRC were analysed separately. Patient and tumour characteristics (i.e. type of cancer, age, sex, stage of disease, region, period) were described at the time of diagnosis. Patient characteristics of the study population were investigated using the Chi‐squared test. An unpaired t‐test was performed to analyse the number of weekly diagnosed patients per period. Possible confounders were based on available data and literature and included in the regression analysis. For BC, confounding variables in the regression models were age, stage of disease and region. For CRC, confounding variables were age, sex, stage of disease and region.

The lead time of the diagnostic pathway during the COVID‐19 period was compared to the same period before the pandemic (2017–2019) and analysed using Cox Proportional Hazards Regression adjusted for possible confounders. During the first peak, population screening was temporarily halted and the number of infections was not equally spread over the Netherlands. To determine possible differences in the magnitude of factors influencing the lead time during the first peak, Cox regression was performed to compare these factors during the first peak in 2020 to 2017–2019.

Data were analysed using Stata version 17.0. A two‐sided *p*‐value of <0.05 was considered statistically significant.

## 3 RESULTS

### 3.1 Research population

**FIGURE 1:**
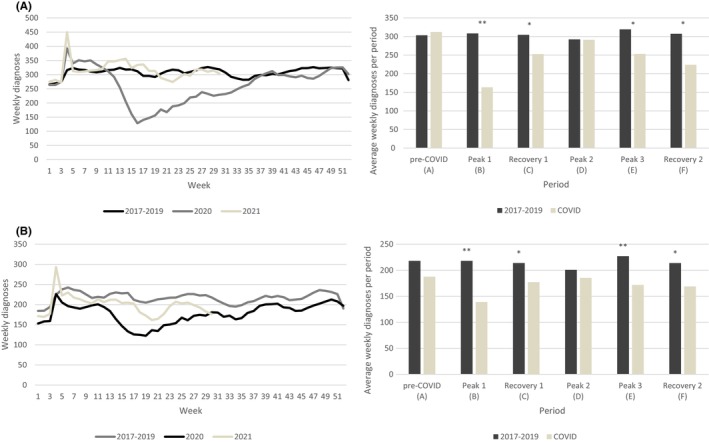
Breast cancer (A) and colorectal cancer (B) diagnoses (average diagnoses per year in periods 2017–2019). Period A (pre‐COVID), weeks 1–11 (2020); Period B (peak 1), weeks 12–20 (2020); Period C (recovery 1), weeks 21–41 (2020); Period D (peak 2), weeks 42–53 (2020); Period E (peak 3), weeks 1–20 (2021); Period F (recovery 2), weeks 21–30 (2021). *Significant difference between periods (*p* < 0.05). **Significant difference between periods (*p* < 0.001).

In total, 71,159 patients with BC and 48,900 patients with CRC were included. The baseline characteristics of patients with BC and CRC are shown in Tables 1 and 2, respectively. The number of diagnosed patients with BC significantly decreased during the first peak, first recovery, third peak and second recovery. CRC diagnoses significantly decreased during all periods excluding the second peak. The number of diagnosed patients had the largest decrease during the first peak in 2020, being 47% and 36% for BC (Figure 1A) and CRC (Figure 1B) respectively. During the pandemic, proportionally fewer patients in the screening age were diagnosed and the proportion of patients with a higher stage increased (Tables 1 and 2). Since stage differed during the pandemic, an additional forward stepwise regression was performed to reveal the effect of stage in the model. The effect of stage on the outcome of lead time and diagnostic procedures performed was strong, therefore stage was included in the final model.

**TABLE 1:** Patient characteristics of breast cancer patients divided per period (*n* = 71,159).

		Period A 2017–2019	Period A pre‐COVID		Period B 2017–2019	Period B peak 1		Period C 2017–2019	Period C recovery 1		Period D 2017–2019	Period D peak 2		Period E 2017–2019	Period E peak 3		Period F 2017–2019	Period F recovery 2	
		*N* (%)	*N* (%)	*p*‐value	*N* (%)	*N* (%)	*p*‐value	*N* (%)	*N* (%)	*p*‐value	*N* (%)	*N* (%)	*p*‐value	*N* (%)	*N* (%)	*p*‐value	*N* (%)	*N* (%)	*p*‐value
Patients	*N*	10014	3435		8329	1472		19189	5309		10527	3543		19162	6389		9222	2952	
Age (years)	<50	2049 (20)	637 (19)	0.007	1590 (19)	394 (27)	<0.001	3648 (19)	1147 (22)	<0.001	1731 (16)	588 (17)	0.915	3820 (20)	1154 (18)	0.001	1820 (20)	465 (16)	<0.001
	50–75	6379 (64)	2191 (64)		5378 (65)	799 (54)		12213 (64)	3059 (58)		7126 (68)	2385 (67)		12251 (64)	4113 (64)		5788 (63)	1940 (66)	
	>75	1586 (16)	607 (18)		1361 (16)	279 (19)		3328 (17)	1103 (21)		1670 (16)	570 (16)		3091 (16)	1122 (18)		1614 (18)	547 (19)	
Stage of disease	Stage 0	1200 (12)	419 (12)	0.896	965 (12)	112 (8)	<0.001	2141 (11)	488 (9)	<0.001	1356 (13)	404 (11)	0.015	2251 (12)	681 (11)	0.100	1019 (11)	331 (11)	0.860
	Stage I	4139 (41)	1388 (40)		3386 (41)	507 (34)		7933 (41)	1904 (36)		4436 (42)	1450 (41)		7855 (41)	2649 (41)		3780 (41)	1187 (40)	
	Stage II	3245 (32)	1138 (33)		2788 (33)	555 (38)		6467 (34)	2007 (38)		3372 (32)	1192 (34)		6325 (33)	2166 (34)		3143 (34)	1014 (34)	
	Stage III	899 (9)	313 (9)		769 (9)	181 (12)		1694 (9)	569 (11)		864 (8)	295 (8)		1736 (9)	550 (9)		809 (9)	250 (8)	
	Stage IV	486 (5)	167 (5)		389 (5)	102 (7)		881 (5)	325 (6)		470 (4)	190 (5)		916 (5)	317 (5)		431 (5)	149 (5)	
	Stage X	45 (0)	10 (0)		32 (0)	14 (1)		73 (0)	16 (0)		29 (0)	12 (0)		79 (0)	26 (0)		40 (0)	21 (1)	
Region	Middle	1959 (20)	722 (21)	0.001	1685 (20)	286 (19)	0.140	3778 (20)	988 (19)	0.001	2174 (21)	711 (20)	0.141	3797 (20)	1240 (19)	0.003	1771 (19)	589 (20)	0.141
	Middle east	862 (9)	348 (10)		732 (9)	160 (11)		1736 (9)	530 (10)		952 (9)	371 (10)		1673 (9)	656 (10)		866 (9)	278 (9)	
	North	1068 (11)	323 (9)		891 (11)	155 (11)		2189 (11)	521 (10)		1097 (10)	357 (10)		2044 (11)	632 (10)		1099 (12)	313 (11)	
	South	2321 (23)	828 (24)		1938 (23)	328 (22)		4342 (23)	1202 (23)		2385 (23)	782 (22)		4432 (23)	1498 (23)		2018 (22)	691 (23)	
	West	3803 (38)	1214 (35)		3083 (37)	543 (37)		7144 (37)	2068 (39)		3919 (37)	1322 (37)		7215 (38)	2363 (37)		3468 (38)	1081 (37)	
	Unknown	1 (0)	0 (0)		0 (0)	0 (0)		0 (0)	0 (0)		0 (0)	0 (0)		1 (0)	0 (0)		0 (0)	0 (0)	

*Note*: Period A (pre‐COVID), weeks 1–11 (2020); Period B (peak 1), weeks 12–20 (2020); Period C (recovery 1), weeks 21–41 (2020); Period D (peak 2), weeks 42–53 (2020); Period E (peak 3), weeks 1–20 (2021); Period F (recovery 2), weeks 21–30 (2021); Pathological stage X, unknown. *p*‐value: Calculated excluding missing values, using the Chi‐squared test to compare the proportion of patients diagnosed in 2020/2021 to 2017–2019.

**TABLE 2:** Patient characteristics of colorectal cancer patients divided per period (*n* = 48,900).

		Period A 2017–2019	Period A pre‐ COVID		Period B 2017–2019	Period B peak 1		Period C 2017–2019	Period C recovery 1		Period D 2017–2019	Period D peak 2		Period E 2017–2019	Period E peak 3		Period F 2017–2019	Period F recovery 2	
		*N* (%)	*N* (%)	*p*‐value	*N* (%)	*N* (%)	*p*‐value	*N* (%)	*N* (%)	*p*‐value	*N* (%)	*N* (%)	*p*‐value	*N* (%)	*N* (%)	*p*‐value	*N* (%)	*N* (%)	*p*‐value
Patients	*N*	7195	2064		5885	1252		13469	3718		7228	2252		13620	4037		6412	1800	
Age (years)	<55	719 (10)	243 (12)	<0.001	618 (11)	168 (13)	<0.001	1389 (10)	491 (13)	<0.001	728 (10)	257 (11)	0.134	1394 (10)	468 (12)	0.026	682 (11)	183 (10)	0.132
	55–75	4165 (58)	1093		3391 (58)	654 (52)		7577 (56)	1823 (49)		4060 (56)	1267		7881 (58)	2337 (58)		3581 (56)	968 (54)	
	>75	2311 (32)	728 (35)		1876 (32)	430 (34)		4503 (33)	1404 (38)		2440 (34)	728 (32)		4345 (32)	1232 (31)		2149 (34)	649 (36)	
Sex	Male	4055 (56)	1169	0.822	3281 (56)	692 (55)	0.756	7425 (55)	1995 (54)	0.111	3980 (55)	1167	0.007	7645 (56)	2238 (55)	0.436	3534 (55)	937 (52)	0.021
	Female	3140 (44)	895 (43)		2604 (44)	560 (45)		6044 (45)	1723 (46)		3248 (45)	1085		5975 (44)	1799 (45)		2878 (45)	863 (48)	
Stage of disease	Stage I	1927 (27)	563 (27)	0.534	1502 (26)	279 (22)	<0.001	3486 (26)	827 (22)	<0.001	1942 (27)	587 (26)	0.192	3588 (26)	1056 (26)	0.783	1662 (26)	450 (25)	0.319
	Stage II	1699 (24)	477 (23)		1488 (25)	283 (23)		3313 (25)	916 (25)		1753 (24)	572 (25)		3312 (24)	976 (24)		1569 (24)	470 (26)	
	Stage III	2240 (31)	616 (30)		1776 (30)	373 (30)		4079 (30)	1157 (31)		2166 (30)	641 (28)		4168 (31)	1249 (31)		1929 (30)	525 (29)	
	Stage IV	1225 (17)	373 (18)		1014 (17)	303 (24)		2376 (18)	764 (21)		1254 (17)	424 (19)		2333 (17)	723 (18)		1145 (18)	345 (19)	
	Stage X	104 (1)	35 (2)		105 (2)	14 (1)		215 (2)	54 (1)		113 (2)	28 (1)		219 (2)	33 (1)		107 (2)	10 (1)	
Region	Middle	1372 (19)	410 (20)	0.073	1085 (18)	266 (21)	0.175	2564 (19)	710 (19)	0.020	1338 (19)	431 (19)	0.325	2572 (19)	781 (19)	0.536	1187 (19)	346 (19)	0.048
	Middle east	687 (10)	214 (10)		550 (9)	110 (9)		1206 (9)	391 (11)		653 (9)	221 (10)		1292 (9)	366 (9)		578 (9)	177 (10)	
	North	793 (11)	184 (9)		650 (11)	123 (10)		1444 (11)	368 (10)		760 (11)	255 (11)		1503 (11)	467 (12)		712 (11)	156 (9)	
	South	1794 (25)	512 (25)		1445 (25)	298 (24)		3345 (25)	947 (25)		1883 (26)	548 (24)		3374 (25)	959 (24)		1574 (25)	454 (25)	
	West	2549 (35)	744 (36)		2153 (37)	454 (36)		4906 (36)	1300 (35)		2593 (36)	796 (35)		4877 (36)	1459 (36)		2359 (37)	664 (37)	
	Unknown	0 (0)	0 (0)		2 (0)	1 (0)		4 (0)	2 (0)		1 (0)	1 (0)		2 (0)	5 (0)		2 (0)	3 (0)	

*Note*: Period A (pre‐COVID), weeks 1–11 (2020); Period B (peak 1), weeks 12–20 (2020); Period C (recovery 1), weeks 21–41 (2020); Period D (peak 2), weeks 42–53 (2020); Period E (peak 3), weeks 1–20 (2021); Period F (recovery 2), weeks 21–30 (2021); Pathological stage X, unknown. *p*‐value: Calculated excluding missing values, using the Chi‐squared test to compare the proportion of patients diagnosed in 2020/2021 to 2017–2019.

### 3.2 Diagnostic procedures

#### 3.2.1 Breast cancer

**FIGURE 2:**
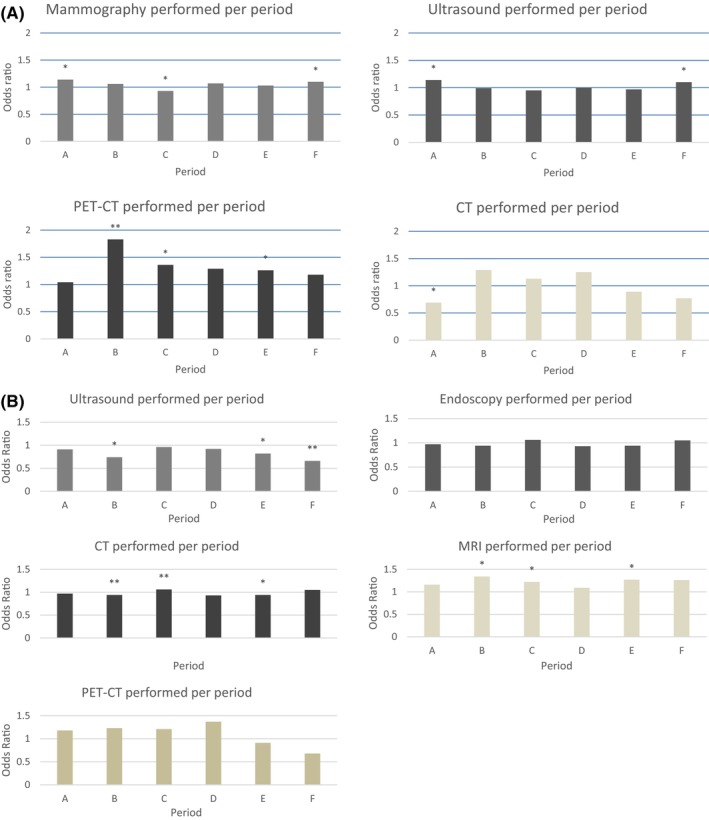
Odds of receiving diagnostic procedure for breast cancer (A) and colorectal cancer (B) per patient. *Period A (pre‐COVID), weeks 1–11 (2020); Period B (peak 1), weeks 12–20 (2020); Period C (recovery 1), weeks 21–41 (2020); Period D (peak 2), weeks 42–53 (2020); Period E (peak 3), weeks 1–20 (2021); Period F (recovery 2), weeks 21–30 (2021). OR: Odds ratio, was calculated using the logistic regression. *Significant difference between periods (*p* < 0.05). **Significant difference between periods (*p* < 0.001).

BC diagnostic procedures performed per patient are shown in Figure 2A. Adjusted for age, stage and region, the percentage of patients who received mammography was significantly lower during the first recovery (from 86% to 84%) and the odds declined (OR = 0.93 [0.87, 0.99] and *p* = 0.017) and was significantly higher (from 86% to 87%) during the pre‐COVID period (OR = 1.14 [1.05, 1.24] and *p* = 0.001) and second recovery (OR = 1.10 [1.00, 1.20] and *p* = 0.039). The percentage of patients who received ultrasound was significantly higher during the pre‐COVID period (from 92% to 93%, OR = 1.14 [1.04, 1.23] and *p* = 0.003) and second recovery (from 92.6% to 93.4%, OR = 1.10 [1.01, 1.21] and *p* = 0.034). The percentage of patients who received PET‐CT was significantly higher during the first peak (from 2% to 5%, OR = 1.83 [1.32, 2.52] and *p* < 0.001), first recovery (from 2% to 3%, OR = 1.36 [1.10, 1.7] and *p* = 0.005) and third peak (from 2% to 3%, OR = 1.26 [1.02, 1.56] and *p* = 0.031). The percentage of patients who received CT was significantly lower in the pre‐COVID period (from 4% to 3%, OR = 0.69 [0.53, 0.90] and *p* = 0.006).

#### 3.2.2 Colorectal cancer

CRC diagnostic procedures performed per patient are shown in Figure 2B. After adjusting for age, sex, stage and region, the percentage of patients who received ultrasound was significantly lower during the first peak (from 16% to 13%, OR = 0.74 [0.59, 0.93] and *p* = 0.008), third peak (from 15% to 14%, OR = 0.82 [0.72, 0.93] and *p* = 0.003) and second recovery (from 15% to 12%, OR = 0.66 [0.54, 0.81] and *p* < 0.001) compared to the same periods in 2017–2019. For patients who received a CT scan, the percentage was significantly higher during the first peak (from 39% to 52%, OR = 1.38 [1.20, 1.59] and *p* < 0.001), first recovery (from 41% to 47%, OR = 1.25 [1.14, 1.35] and *p* < 0.001 and third peak (from 38% to 43%, OR = 1.14 [1.05, 1.24] and *p* = 0.003). The percentage of patients who received MRI was significantly higher during the first peak (from 7% to 10%, OR = 1.34 [1.03, 1.75] and *p* = 0.029), first recovery (from 7% to 9%, OR = 1.22 [1.04, 1.44] and *p* = 0.017) and third peak (from 7% to 9%, OR = 1.27 [1.08, 1.49] and *p* = 0.004). The percentage of patients who received endoscopy and PET‐CT was not significantly different in any period.

### 3.3 Lead times

#### 3.3.1 Breast cancer

**FIGURE 3:**
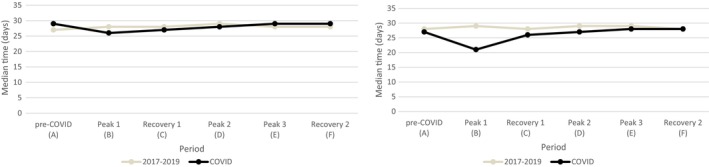
Lead times of the diagnostic pathway for breast (left) and colorectal cancer (right). COVID: Period A (pre‐COVID), weeks 1–11 (2020); Period B (peak 1), weeks 12–20 (2020); Period C (recovery 1), weeks 21–41 (2020); Period D (peak 2), weeks 42–53 (2020); Period E (peak 3), weeks 1–20 (2021); Period F (recovery 2), weeks 21–30 (2021).

The median time of the diagnostic pathway of BC is shown in Figure 3. The hazard ratios and *p*‐values are shown in Table 3. For 21,463 BC patients, the date of diagnosis is used as the date of first diagnostic procedure. After adjustment for age, stage and region, the lead time of the diagnostic pathway of BC significantly decreased during the first peak with 2 days (HR = 1.21 [1.14, 1.28]), first recovery with 1 day (HR = 1.08 [1.04, 1.11]) and second peak with 1 day (HR = 1.14 [1.10, 1.19]). The median lead time significantly increased during the pre‐COVID period with 2 days (HR = 0.83 [0.80, 0.86]) and during the third peak and second recovery with 1 day (both HR = 0.93 [0.89, 0.97]).

**TABLE 3:** Time to therapy of diagnostic pathway corrected for confounders: result of the Cox regression analysis.

Period	Time to therapy—Hazards ratio [95% CI]					
	A (pre‐COVID)	B (peak 1)	C (recovery 1)	D (peak 2)	E (peak 3)	F (recovery 2)
Breast cancer	0.83 [0.8, 0.86]**	1.21 [1.14, 1.28]**	1.08 [1.04, 1.11]**	1.14 [1.1, 1.19]**	0.91 [0.89, 0.94]**	0.93 [0.89, 0.97]*
Colorectal cancer	1.01 [0.96, 1.06]	1.43 [1.35, 1.53]**	1.11 [1.07, 1.15]**	1.11 [1.06, 1.16]**	1.04 [1, 1.08]*	0.96 [0.91, 1.02]

*Note*: Period A (pre‐COVID), weeks 1‐11 (2020); Period B (peak 1), weeks 12–20 (2020); Period C (recovery 1), weeks 21–41 (2020); Period D (peak 2), weeks 42–53 (2020); Period E (peak 3), weeks 1–20 (2021); Period F (recovery 2), weeks 21–30 (2021); Corrected for age, gender (only colorectal cancer), stage and region.

* Significant difference between periods *p* < 0.05.

** Significant difference between periods *p* < 0.001.

#### 3.3.2 Colorectal cancer

The median time of the diagnostic pathway of CRC is shown in Figure 3. The hazard ratios and *p*‐values are shown in Table 3. For 19,841 CRC patients, the date of diagnosis is used as the date of the first diagnostic procedure. After adjustment for age, sex, stage and region, the lead time of the diagnostic pathway of CRC significantly decreased during the first peak with 8 days (HR = 1.43 [1.35, 1.53]), first recovery with 2 days (HR = 1.11 [1.07, 1.15]) and second peak with 2 days (HR = 1.11 [1.06, 1.16]).

#### 3.3.3 Factors influencing the lead time during the first peak

During the first peak, differences in factors were found for BC and CRC. For BC, a larger decrease in median time to therapy was found for patients in the age group >75 and stage 4 compared to the other categories. For CRC, the results were the opposite. The decrease in lead time for patients aged >75 and patients in stage 4 was smaller compared to other categories. No notable differences in impact were found between regions in terms of the lead time of the diagnostic pathway for both BC (Table 4) and CRC (Table 5).

**TABLE 4:** Factors influencing the lead times of diagnostic pathway during the period of the first peak for 2017–2019 and 2020 separate for breast cancer (*n* = 8329 and *n* = 1472, respectively).

Breast cancer—diagnosis to therapy				
Period B (peak 1)	2017–2019		2020	
	Hazard ratio [95% CI]	Median time (days)	Hazard ratio [95% CI]	Median time (days)
Age cat				
50–75	1	29	1	27
<50	0.9 (0.85–0.95)**	30	0.97 (0.86–1.1)	27
>75	1.25 (1.18–1.33)**	25	1.47 (1.28–1.69)**	21
Stage				
0	0.68 (0.64–0.73)**	34	0.81 (0.66–0.99)*	29
1	1	28	1	26
2	0.98 (0.93–1.03)	28	1.04 (0.92–1.18)	25
3	1.02 (0.94–1.1)	28	0.96 (0.81–1.14)	26
4	1.15 (1.03–1.27)*	28	1.45 (1.17–1.8)*	18
Region				
Middle	1.01 (0.95–1.08)	28	0.98 (0.83–1.14)	23
Middle east	1.06 (0.97–1.16)	28	0.81 (0.67–0.98)*	28
North	1.01 (0.93–1.1)	29	0.81 (0.67–0.99)*	27
South	1	28	1	23
West	0.94 (0.89–1)*	29	0.79 (0.69–0.91)*	27

*Note*: Period B (peak 1), week 12–20 (2020).

* Significant difference between periods *p* < 0.05.

** Significant difference between periods *p* < 0.001.

**TABLE 5:** Factors influencing the lead times of diagnostic pathway during the period of the first peak for 2017–2019 and 2020 separate for colorectal cancer (*n* = 5885 and *n* = 1252, respectively).

Colorectal cancer ‐ time to therapy				
Period B (peak 1)	2017–2019		2020	
	Hazard ratio [95% CI]	Median time (days)	Hazard ratio [95% CI]	Median time (days)
Age cat				
55‐75	1	30	1	21
<55	1.21 (1.11–1.32)**	27	1.28 (1.08–1.52)*	19
>75	1.01 (0.95–1.07)	30	0.83 (0.74–0.95)*	23.5
Sex				
Female	1	29	1	21
Male	0.91 (0.87–0.96)*	30	0.99 (0.89–1.11)	22
Stage				
1	1	29	1	19
2	1 (0.93–1.07)	31	0.92 (0.78–1.09)	21
3	1.02 (0.96–1.1)	30	0.89 (0.76–1.04)	23
4	1.02 (0.95–1.11)	27	0.85 (0.72–1)	22
Region				
Middle	0.9 (0.83–0.97)*	28	1.02 (0.87–1.21)	20
Middle east	0.85 (0.77–0.94)*	32.5	0.76 (0.61–0.96)*	24
North	0.81 (0.74–0.89)**	33.5	0.78 (0.63–0.96)*	26
South	1	28	1	21
West	0.92 (0.86–0.98)*	29	0.86 (0.74–1)	22

*Note*: Period B (peak 1), week 12–20 (2020).

* Significant difference between periods *p* < 0.05.

** Significant difference between periods *p* < 0.001.

## 4 DISCUSSION

As a result of the measures taken to control the spread of COVID‐19, that is, the temporary halt of the national screening program and the advice to visit the GP only in case of severe complaints, the number of BC and CRC diagnoses decreased. Compared to the years 2017 to 2019, the frequency of hospital diagnostic procedures decreased for both tumour types and there was a shorter time to therapy.

Stage was important to take into account in the model due to the increased proportion of patients diagnosed at a higher stage. An additional forward stepwise regression was performed to reveal the effect of stage in the model. For lead time and several diagnostic procedures, the stage was added to the model as the first and most important variable. The effect of stage on the outcome was strong and therefore included in the final model.

The lower number of BC and CRC diagnoses during the pandemic, mainly in the lower stages, was to be expected due to the halt of the screening program in which low stages are generally detected. Besides, for CRC, a decreasing trend in the number of CRC diagnoses has been present for the past years as a result of the nationwide screening program.^16^ However, as in other studies, the decrease in number of diagnoses during the first peak of the pandemic was larger compared to the other COVID‐19 periods.^8^, ^16^

There were significant differences in the diagnostic procedures performed per patient. For BC, the percentage of patients who received mammography was significantly lower during the COVID‐19 period and the percentage of patients who received PET‐CT was significantly higher. The percentage of patients with CRC who received ultrasound was significantly lower during the pandemic and the percentage of patients who received CT and MRI was significantly higher.

The higher proportion of PET‐CTs, CTs and MRIs possibly reflects the increased proportion of patients diagnosed at a higher stage, which is associated with more symptoms. Therefore, different diagnostic procedures were performed compared to preceding periods in which more asymptomatic patients with lower‐stage disease were predominant. Stage was important to take into account in the model due to the increased proportion of patients diagnosed at a higher stage.

This study showed that lead time of the diagnostic pathway of BC and CRC was significantly shorter during the pandemic, which was consistent with other studies.^10^, ^14^, ^17^ A shortened time to therapy, probably the result of fewer referred patients with cancer to the hospital and the prioritising of oncologic care, possibly allowed diagnostics to be performed more quickly. The decrease may also be explained by a change from an initial surgical treatment^7^ to a hormonal treatment or radiotherapy, which led to an earlier start of therapy. Thirdly, the decrease may be due to the fact that, relatively seen, patients presented with symptoms and a larger tumour burden related to a higher stage, therefore, diagnosis is easier and treatment can start earlier.

Differences in age categories and stage of disease were found. For BC, the largest decrease in time to therapy was seen among the elderly or a higher stage. For CRC, the time to therapy for the elderly or a higher stage was less decreased in comparison with other patients. This may be due to the fact that co‐morbid diseases in elderly people have more impact on the treatment options in patients diagnosed with CRC than in patients diagnosed with BC.

A limitation of this study was that the percentage of patients with BC and CRC who received a biopsy and the percentage of patients with BC who received an MRI were not completely available and therefore these diagnostic procedures could not be included in the current analysis. However, this does not affect the results of this study.

## 5 CONCLUSIONS

In conclusion, the COVID‐19 pandemic significantly impacted the diagnostic pathway of patients both with BC and CRC. The impact was mainly observed in 2020, particularly during the first peak of COVID infections. There was a drop in number of diagnoses resulting from the temporary halt of population screening. The percentage of patients who received diagnostic procedures for early stage tumours decreased (i.e. less mammography for BC, less endoscopy for CRC), and a shortened time to therapy was observed possibly related to the alterations in first therapy. Diagnostics regarding BC and CRC in 2021 were comparable with the pre‐COVID period (period A), which means that care returned to the same standards. The long‐term effects of these findings on patient outcomes are not known yet and this will be the subject of future research.

## AUTHOR CONTRIBUTIONS

**Wouter Wolfkamp:** Conceptualization (lead); data curation (lead); formal analysis (lead); investigation (lead); methodology (lead); project administration (lead); visualization (lead); writing – original draft (lead); writing – review and editing (lead). **Joyce Meijer:** Data curation (supporting); formal analysis (supporting); investigation (supporting); methodology (supporting); visualization (supporting); writing – original draft (supporting); writing – review and editing (supporting). **Jolanda C. van Hoeve:** Data curation (supporting); formal analysis (supporting); funding acquisition (supporting); investigation (supporting); methodology (supporting); resources (supporting); software (supporting); validation (equal); visualization (supporting); writing – original draft (supporting); writing – review and editing (supporting). **Felice van Erning:** Validation (equal); writing – original draft (supporting); writing – review and editing (supporting). **Lioe‐Fee de Geus‐Oei:** Validation (equal); writing – original draft (supporting); writing – review and editing (supporting). **Ignace de Hingh:** Validation (equal); writing – original draft (supporting); writing – review and editing (supporting). **Jeroen Veltman:** Investigation (supporting); methodology (supporting); validation (equal); visualization (supporting); writing – original draft (supporting); writing – review and editing (supporting). **Sabine Siesling:** Data curation (supporting); formal analysis (supporting); funding acquisition (lead); investigation (supporting); methodology (supporting); resources (lead); software (lead); validation (equal); visualization (supporting); writing – original draft (supporting); writing – review and editing (supporting).

## FUNDING INFORMATION

ZonMw, project number: 10430022010014.

## CONFLICT OF INTEREST STATEMENT

J.V.: President Dutch college of breast imaging; L.G.: NWO‐NWA payments were made; I.H.: Research funding: Roche, RanD. The other authors declare that they have no competing interests.

## ETHICS STATEMENT

The Committee of Privacy of the NCR agreed upon the data application (application number K22.057) and the DHD application (application no. L21.029). Hospitals were asked for permission to use the data on diagnostic procedures which was obtained for 69 out of 76 hospitals.

## Data Availability

The datasets generated during and/or analysed during the current study are not publicly available due to privacy regulations but are available from the corresponding author on reasonable request or by sending a request to the Netherlands Comprehensive Cancer Organisation (gegevenaanvraag@iknl.nl).
